# Inoculation of Native Arbuscular Mycorrhizae and *Bacillus subtilis* Can Improve Growth in Vegetable Crops

**DOI:** 10.1155/2024/9226715

**Published:** 2024-05-07

**Authors:** Sara Gebreslassie, Mulissa Jida, Mariana Laura Puente, Fernanda Covacevich, Zerihun Belay

**Affiliations:** ^1^Department of Applied Biology, Adama Science and Technology University, Adama, Ethiopia; ^2^Bio and Emerging Technology Institute, Ras Biru Street, Near TemenJa Yaj, Addis Ababa, Ethiopia; ^3^Instituto Nacional de Tecnología Agropecuaria (INTA), Instituto de Microbiología y Zoología Agrícola, Buenos Aires, Argentina; ^4^Consejo Nacional de Investigaciones Científicas y Técnicas, Instituto de Investigaciones en Biodiversidad y Biotecnología, Mar del Plata, Argentina

## Abstract

Arbuscular mycorrhizal fungi (AMF) and some rhizobacteria are known as plant growth-promoting microorganism (PGPM) as they play significant roles in improving soil fertility structure, plant nutrition, growth, and health. However, little is known about the PGPM potential of AMF and rhizobacteria native to the Rift Valley and highland regions of Ethiopia. Hence, this study aimed to investigate the PGPM effect of single and co-inoculation of AMF and the *Bacillus subtilis* ALCR46 strain, on tomato (*Lycopersicum esculentum* L.), onion (*Allium cepa* L.), and squash (*Cucurbita pepo* L.) plants. The experimental setup was a randomized complete block design with three replications of the following treatments: (i) inoculation with a consortium of AMF, (ii) co-inoculation with a consortium of AMF and the *Bacillus subtilis*, (iii) inoculation with *Rhizophagus clarus*, (iv) co-inoculation with *R. clarus* and *B. subtilis*, (v) inoculation with *B. subtilis*, (vi) plants without inoculation (negative control), and (vii) plants treated with chemical fertilizer (positive control). Plants were maintained in a greenhouse for 60 days, and after harvest, plant growth parameters, percentage of AMF root colonization, and spore number were analyzed. The result shows that the growth of crops significantly increased by co-inoculation with the consortium of AMF and *B. subtilis*. AMF spore density and root colonization rate were also increased in co-inoculated plants. Highest root colonization, spore number, and mycorrhizal dependency were observed in *A. cepa*. Our results suggest that there is a synergistic effect between the AMF and *B. subtilis* ALCR46, and between AMF inoculants. However, the application of present findings under field conditions is required to be confirmed by further studies.

## 1. Introduction

Vegetables are essential for food and nutrition security. They are key sources of macro-and micronutrients, including vitamins, trace minerals, dietary fiber, phytochemicals, and many other classes of biologically active compounds [1]. They are involved in different biological activities, such as stimulation of the immune system, reduction of platelet aggregation, modulation of cholesterol synthesis and hormone metabolism, reduction of blood pressure, and antioxidant, antibacterial, and antiviral effects [2].

The production of vegetables in Africa, as well as in Ethiopia, is lagging far behind the worldwide average (1089 million tons) [3], due to a lack of advanced agricultural technologies and yield losses caused by the extreme sensitivity to biotic and abiotic factors [4]. Most vegetables are produced using synthetic fertilizers and pesticides, which involve high production costs and also have adverse effects on humans, animals, and the environment [5]. To solve these problems, the urgent development of more cheap and sustainable strategies is required [6]. Several technical and technological innovations are proposed to improve agricultural production systems in the framework of a sustainable agriculture that includes a significant reduction in agrochemicals. A promising practice would be the use of soil microorganisms called plant growth-promoting microorganism (PGPM) that enhance plant growth and increase tolerance to disease and extreme environmental conditions [7].

Among soil PGPM, arbuscular mycorrhizal fungi (AMF) and, plant growth-promoting rhizobacteria (PGPR), are the most promising to improve the sustainability of production systems through a significant reduction in agrochemicals. AMF form a symbiotic relationship with roots of 80–90% of terrestrial plant species, including cereals, legumes, and horticultural plants [8]. The AMF, through inner colonization of plant roots, benefit the host by increasing nutrient uptake, mainly N and P. Moreover, they improve drought, salinity, heavy metals, and pathogen resistance of the host [9, 10]. Recently, Lee et al. [11] and Sagar et al. [12] confirmed that also several bacterial species mostly associated with the plant rhizosphere are found to be beneficial for plant growth, yield, and crop quality. Some of these organisms known as PGPR are beneficial microorganisms capable of improving plant growth through the solubilization/mineralization/biological fixation of nutrients and/or through biotic/abiotic stress reduction could contribute to crop productivity/health while maintaining environmental sustainability [5]. Various studies reported that co-inoculation of AMF and the PGPR exerted positive effects on the growth and yield of several crops,enhances nutrient uptake and productivity compared to single inoculation under both normal and stressed conditions [11, 12]. They concluded that co-inoculation was more effective to ameliorate plant stresses and to improve growth; furthermore, they showed higher AMF spore density and AMF root colonization as compared to single AMF inoculation. Among PGPRs, it is highlighted *Bacillus,* which is a cosmopolitan bacterium inhabiting rhizosphere/roots-associated soil that establishes association with roots. It is a spore former bacterium with recognized saprophytic ability and competitiveness and can survive in the soil for long period of time under harsh environmental conditions. *Bacillus* spp. assist plants in their defense against pathogen attack and also enhance stress tolerance by inducing the expression of stress response genes, and producing phytohormones and stress-related metabolites [7].

Despite the fact that AMF commercial bioinoculants are available on the market, research has shown that these inoculants sometimes did not establish in the soil trophic network and did not increase the biomass of native plants; instead, some of them increased the growth of invading plant species while decreasing the growth of native species [13]. Considering the potential of AMF and *Bacillus*, a promising strategy is to explore native strains and establish candidates for the formulation of bioinoculants. Williams et al. [14] made a comparative study on the effect of indigenous AMF inoculums obtained from environmental samples in the Mackenzie Basin, New Zealand, with commercially available AMF on growth and survival of *Podocarpus cunninghamii* (mountain tōtara). They reported that plants treated with indigenous and forest AMF showed significantly a greater survival and growth than those treated with commercial AMF. In addition, a field trial conducted with various combinations of PGPR and AMF at the Central University of Punjab (India) showed that the consortia based on native and non-native PGPR (*Bacillus* spp.) and AMF performed better in terms of nutrient content in wheat grain tissue and yield-related traits compared with chemical fertilizer treated and untreated control [15]. The same authors conducted an open greenhouse pot experiment, based on the evaluation of potentiality of wheat inoculation with native and non-native bacteria alone or in co-inoculation with AMF. They showed that combined inoculation with native PGPR and AMF showed higher yield-related parameters and nutrient content of wheat grains compared to single inoculation with non-native bacteria [16].

Although some studies have documented the diversity and richness of the AMF in soils under different land use systems in Ethiopia [17–20], there is no information on the PGPM potential of co-inoculation with *Bacillus* and AMF indigenous to enhance plant growth. This study is focused on the potentiality of single and dual inoculation with a consortium with AMF and a *Bacillus* indigenous from Ethiopia to increase the growth of agronomically valuable crops.

## 2. Materials and Methods

### 2.1. Study Sites Characteristics and Soil Sample Collection

Soil samples were collected from selected areas of lowland (at the Central Rift Valley) and highland regions of Ethiopia. The Central Rift Valley region includes the Batu and Bishoftu woody grassland in which naturally different acacia tree species are dominant [21]. The region has a mean annual temperature and rainfall of 20°C and 816 mm, respectively. The highland studied region was Sululta, which is located in the central part of Ethiopia, Oromia Special Zone 23 km from Addis Ababa to the north, geographically extends from 9°30′00″N to 9°12′15″N latitude and 38°42′0″E to 38°46′45″ E longitude [22].

Seven samples of soil near to roots of acacia trees were collected in January 2021 as follows: samples of soil near to roots of *Acacia seyal*, *Acacia tortilis*, and *Acacia saligna* were collected at Batu and Bishoftu (lowlands) that resulted in six composite soil samples, and one composite sample of soil near to roots of *Acacia abyssinica* was collected at Sululta (highlands). Each sample was collected near to roots of the acacia species that were randomly selected from areas of approximately 100 m^2^. Each sample resulted in the pooled of about 3 kg of soil of each subsample that was collected into a depth of 30 cm. The samples were collected in alcohol-sterilized plastic bags, air-dried, and stored at room temperature until further analysis was conducted. Spore abundance and physico-chemical parameters of the soil samples were determined before the establishment of trap cultures (Table 1).

### 2.2. Establishment of Trap Cultures

To obtain healthy and infective AMF spores and propagules for inoculum production, trap cultures were established in the greenhouse at ASTU following the method of INVAM (https://invam.caf.wvu.edu). Briefly, each soil sample was thoroughly mixed with autoclaved sand (1 : 1 v/v); then, about 3 kg of the mixture of fresh soil: washed and autoclaved sand was transferred to plastic pots. Seeds of sorghum ([*Sorghum bicolor* (L.) Moench] “Melkam” cultivar), known as mycotrophic crop for its ability to induce high spore density, diversity, and species richness (INVAM https://invam.caf.wvu.edu), were provided by the Melkassa Agricultural Research Center (MRC) and used as a trap plant. Surface-sterilized seeds were over seeded in each plastic pot filled with the soil: sand mixture and covered with washed and autoclaved sand. Pots were irrigated daily as needed. All seedlings were grown in a greenhouse under natural light and temperature conditions during four months. Watering was reduced during the final two weeks after harvest to maximize spore production. After that, plants were cut near the base, and the cultures (multiplied AMF spore and mycelia, AMF-colonized roots, and accompanying microbiota) were air-dried and stored in zipped plastic bags at room temperature for 30 days before inoculation experiment INVAM (https://invam.caf.wvu.edu).

### 2.3. Determination of AMF Root Colonization and Spore Density of Trap Cultures

Young and fresh roots of the trap cultures were washed thoroughly under tap water to remove soil particles and other organic debris and stored at 4°C in 50% ethanol to determine the percentage of AMF root colonization. Cleaning and staining of roots were conducted according to the techniques adopted from Brundrett et al. [23]. Quantification of AMF root colonization was performed using the magnified intersection method of Mc Gonigle et al. [24] under a compound light microscope at a magnification of 200x. Accordingly, 150 intersections were observed for each sample. The presence of AMF hyphae, vesicles, and arbuscules was recorded. Furthermore, AMF spores were extracted from the trap culture soil by wet sieving and decanting method [25] followed by centrifugation in water and in a 50% sucrose solution [23]. Sieve sizes of 500 *μ*m, 250 *μ*m, and 53 *μ*m were used for the wet sieving procedure. Spores, spore clusters, and sporocarps obtained from 250 and 53 *μ*m sieves were counted and observed by using a dissecting microscope. Spore abundance resulted in enumeration of spore numbers per gram of dry soil (https://invam.caf.wvu.edu).

### 2.4. Experiment of Plant Growth Promotion after AMF and PGPR Inoculation

An experiment was set up in a randomized complete block design with three replications in a greenhouse in which plant growth promotion of tomato (*Lycopersicum esculentum*) var. Melkashola, onion (*Allium cepa*), and squash (*Cucurbita pepo*) var. Jp-10 was studied against following treatments: T1: a consortium of AMF previously studied [17] and multiplied inoculum (32.4 spores g^−1^ of soil); T2: a consortium of AMF [17] and *Bacillus subtilis* (∼10^8^ CFU mL^−1^); T3: inoculation with AMF*-Rhizophagus clarus* (29 g^−1^ of soil); T4: inoculation with *Rhizophagus clarus* and *B. subtilis*; T5: inoculation with *B. subtilis*; T6: negative control (noninoculated and not fertilized); and T7: positive control (chemical fertilizer). Seeds of tested plants were provided by the Melkassa Agricultural Research Institute (MRC, Ethiopia) and were surface sterilized with 0.5% sodium hypochlorite solution for 15 minutes and allowed to germinate on a 0.75% (w/v) water agar for 3 days at 25°C before planting. Then, two germinated seeds were sown at 2 cm depth in each plastic pot. Soil for fill pots of experiment was collected from Holeta, west Showa zone, and physical and chemical characteristics before experiment were as follows: availability of phosphorus (P) (6.44 ppm), total nitrogen (TN) (1.66%), organic carbon (OC) (1.549%), pH (4.75), and electric conductivity (EC) (0.059 ds/m) [26]. Pots for plants growth were filled with 4 kg of autoclaved growth substrates (mixture of field soil and washed sand, 1 : 1 v/v). Inoculum of AMF consisted of 100 g of previously multiplied inoculum (consortium of AMF, 32.4 spores g^−1^ soil) or *R. clarus* (29 g^−1^ of soil), which was provided by the Department of Biology, Hawassa University (Ethiopia). Both inoculums consisted of infected root segments of host trap plants, spores, and extraradical hyphae (INVAM, https://invam.caf.wvu.edu).

Inoculum of PGPR consisted of ∼10^8^ CFU mL^−1^*B*. *subtilis* ALCR46, which were counted through viable plate count method and optical density measurement using a spectrophotometer (SM-1600 Double Beam UV/VIS Spectrophotometer at 600 nm). Strain ALCR46 was previously isolated at Addis Ababa University and reported as plant growth-promoting bacteria [27]. Prior to experiment, inoculum was previously multiplied into 250 mL of nutrient broth and incubated with shaking at 30°C, for 24 h. Inoculation consisted of application on 1 mL of culture suspension at planting and 5 mL of the suspension one week after planting [28]. In the positive control (T7), two fertilization schemes were applied: first at the beginning directly to the soil with 1.32 g plant^−1^ of urea and ammonium phosphate, dibasic [(NH4)_2_HPO_4_ containing 18% nitrogen and 46% phosphorus (P_2_O_5_)], and the second a foliar fertilization with the formulation 12.10.8 (N-P-K) by dissolving 7.5 mL in 3 L of distilled water every 15 days [29]. An equal volume of steam-sterilized inoculum lucking treatments was applied into the negative control, to ensure that trace elements were consistent. All pots were irrigated daily as needed. All seedlings were grown in the greenhouse under natural light and temperature conditions for 60 days.

### 2.5. Mycorrhization and Plant Growth Analysis

The measurement of plant growth parameters was conducted twice at 35- and 60-day intervals after sowing. For this, one seedling from each pot was uprooted at the end of 35 days, and the remaining seedling from each pot was uprooted at the end of the growth season (60 days) for the measurement of growth/mycorrhizae parameters. At each harvest, plant roots from each pot were sampled, cleared and stained and AMF colonization was determined as described before for trap plant cultures. AMF spore abundance occurring in rhizosphere of grown seedlings was examined at the end of the experiment (60 days) by the wet sieving and decanting method followed by centrifugation in water and in a 50% sucrose solution as described before (INVAM, https://invam.caf.wvu.edu). Plant height, number of branches per plant, shoot and root fresh weight, and shoot and root dry weight were recorded at the end of the experiment.

Mycorrhizal dependency (MD) value was determined following the method of Plenchette et al. [30] as follows:

(1) $$\begin{array}{c} \%=\frac{\text{dry mass inoculated plant}-\text{dry mass noninoculated }\left(T6\right)\text{ plant}}{\text{dry mass inoculated plant}}\times 100. \end{array}$$

### 2.6. Data Analysis

All data were subjected to analysis of variance (ANOVA) to determine the significance of the host plants and inoculation treatments, and their interaction with the experimental parameters. Comparisons between means (in three replicates) were performed with Tukey's honestly significant difference (HSD) test at the significance level of *p* ≤ 0.05. The relationships among various parameters were analyzed by using multiple logistic regression tests. The statistical analyses were carried out with the SPSS software package (version 26.0).

## 3. Results

### 3.1. AMF Root Colonization and Spore Density of the Trap Cultures

The average number of AMF spore recorded in the trap cultures ranged from 18.7 to 32.4 spores g^−1^ soil (Table 2). There was a significant difference in AMF spore density among the areas of sampling and rhizosphere of the acacia species. Highest spore densities were recorded from trap culture sampled from the rhizosphere of *A. seyal* in Batu. Lowest AMF spore density (18.7 spores g-1 soil) was found at the trap culture from the soil associated with *A. abyssinica* in the Sululta region. Roots of all trap cultures were colonized by AMF, and root mycorrhizal colonization significantly differed between the sampling areas and host plants (*p* < 0.05) (Table 2). Average values of total colonization ranged between 56.9 and 94.9%. The highest percentage of TC was found in roots of the trap culture in which the AMF of soil associated with roots of *A. seyal* (94.9%) from Batu were multiplied. The lowest percentage of AMF total colonization (TC) was obtained in roots of the trap culture in which the AMF of soil associated with roots of *A. abyssinica* (56.9%) from Sululta were multiplied. Likewise, the lowest and highest arbuscular colonization (AC) and vesicular colonization (VC) were obtained in roots of the trap culture in which the AMF of soil associated with roots of *A. abyssinica* (25.2% and 14.6%) from Sululta and *A. seyal* (45.7%) from Batu and (27.3%) from Bishoftu, respectively, were multiplied.

### 3.2. Plant Growth Responses to AMF and *B. Subtilis* ALCR46 Inoculation

#### 3.2.1. *Lycopersicum esculentum*

The growth parameters of *L. esculentum* are shown in Table 3. The seedlings treated with chemical fertilizer [positive control (T7)] and that received dual inoculation with the consortium of AMF + *B. subtilis* (T2) showed a significantly greater response at all growth parameters over the negative control (T6) treatment. Interestingly, no significant differences were found between the T7 (positive control) and T2 (dual inoculation) for all assessed parameters. Except for the T2, all seedlings treated with microbial inoculations showed significantly lower average values of growth in all parameters when compared to the T7. The average of shoot and root height of seedlings after dual inoculation with *R. clarus* + *B. subtilis* (T4) and also after single inoculation with the consortium of AMF (T1) was lowered by 12.6% and 24.5%, and 18% and 38.7% (for shoot at T4 and T1 and roots at T4 and T1, respectively), as compared to seedlings treated with T7.

Among inoculated plants, those inoculated with the consortium of AMF + *B. subtilis* (T2) showed significant increases in plant growth parameters than plants that received single inoculation. The average shoot and root height and the number of branches of *L. esculentum* inoculated with T2 were increased by 17.4%, 33.5%, and 10%, respectively, compared to that of T1. There was also a significant increase in the average of fresh weight of shoot (36%) and root (63.6%), and dry weight of shoot (111.7%) and root (170%) in T2 treatment compared to the T1 inoculation (Table 3).

The results also showed that the growth potential of *L. esculentum* seedlings inoculated with single ether consortium AMF (T1), *R. clarus* (T3), or *B. subtilis* (T5) was better performed than the negative control (T6). The seedlings treated with T1 inoculation showed the best growth response compared to other single treatments, with a significant difference in most growth parameters. The average root height, fresh weight of shoot and root, and dry weight of shoot and root in the T1 inoculation treatment were increased by 15.5%, 19.2%, 46.7%, 50%, and 42.9%, respectively, compared to T3 (Table 3). However, no significant difference was found in shoot height or the number of branches between T3 and T1.

#### 3.2.2. *Allium cepa*

*Allium cepa* seedlings treated with T2 showed significant increases in most growth parameters compared to the dual inoculation of T4 or single inoculation of T1, T3, or T5 (Table 3). *A. cepa* seedlings inoculated with T2 and T7 significantly showed better growth potential than the other treatments, but plants of treatments T2 and the T7 did not show significant differences in most of growth parameters except in shoot height. The average shoot height, root height, shoot fresh weight, root fresh weight, shoot dry weight, and root dry weight in the T2 were increased by 14.9%, 24.6%, 26.9%, 100%, 100%, and 50%, respectively, compared to the T1 (Table 3). Plants inoculated with T1 showed growth increases in shoot and root fresh weight and shoot and root dry weight of 29.3, 100, 16.7, and 100%, respectively, with respect to plants inoculated with T3 (Table 3). However, there was no significant difference in growth response between the T5 and T6 (Table 3).

#### 3.2.3. *Cucurbita pepo*

Dual inoculation with AMF + *B. subtilis* significantly increased the growth of *C. pepo* compared to noninoculated plants (Table 3). The highest average growth parameters were recorded in the T7, followed by the T2. However, no significant differences were observed between T2 and T7 in all growth parameters (*p* > 0.05). Plants inoculated with T2 showed growth increases in shoot and root fresh weight and shoot and root dry weight of 12%, 47%, 77.2%, and 120%, respectively, with respect to plants inoculated with T1 (Table 3).

Among the single inoculations, the effect of T1 treatment was significantly higher than T3 in root fresh weight and shoot dry weight, but no significant differences were observed in the other parameters. Correspondingly, seedlings treated with T1 significantly showed increases in all growth parameters compared to T5 and T6 (Table 3). The dry weight of shoot and root in T1 increased by 58%, 100%, 192.6%, and 233.3% than in T5 and T6, respectively. However, no significant differences were found in growth parameters between T5 and T6 except in shoot height, shoot fresh, and dry weight in which T5 was higher than T6.

### 3.3. AMF Root Colonization and Spore Density

All roots that received AMF either from the native consortium alone or co-inoculated with *Bacillus* (T1 and T2, respectively) or AMF from the culture of *R. clarus* alone or co-inoculated with *Bacillus* (T3 and T4, respectively) showed characteristic mycorrhizal colonization structures (arbuscules, vesicles, and hyphae), while, as expected, the roots of the seedlings that did not receive AMF did not show evidence of mycorrhizae.

**Figure 1:**
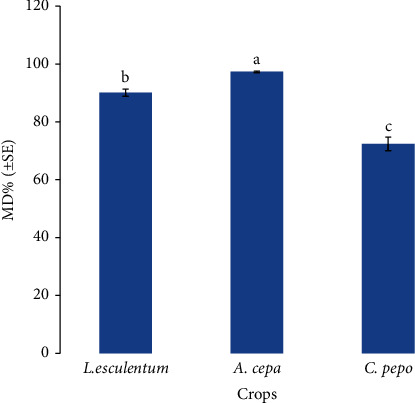
Comparison of mycorrhizal dependency (MD) among crops. Mean values of MD % followed by different letters indicate significant differences among the crops after post hoc Tukey tests (*P* < 0.05).

The total root colonization recorded after 35 days was significantly different among host plants and inoculation treatments (Table 4). In *L. esculentum*, *A. cepa*, and *C. pepo*, the highest TC was found in roots of plants that received dual inoculation of the consortium of AMF and *B. subtilis* (T2), and the lowest in roots of plants that received single inoculation with *R. clarus* (T3). Root colonization progress was evidenced by increases in TC between 35 and 60 days. In this regard, increases in TC between 35 and 60 days in the roots of the T2 plants of 170.7%, 129.2%, and 178%, in *L. esculentum, A. cepa*, and *C. pepo*, respectively, were recorded. Likewise, the highest AMF spore abundance at the substrate at 60 days of plant growth was recorded after the consortium of AMF and *B. subtilis* (T2) followed by dual inoculation with *R. clarus* and *B. subtilis* (T4). In general, the lowest spore abundance was recorded at T3. *A. cepa* was the most mycotrophic host plant, followed by *L. esculentum* in terms of the percentage of AMF root colonization, spore density, and mycorrhizal dependency (Table 4; Figure 1). Furthermore, among inoculated treatments, the inoculation with the consortium of AMF and *B. subtilis* (T2) increased, at 60 days of growth, and on average for all plants studied, the TC of about 30.7%, 85.3%, and 18.5% and the spore abundance of about 67.5%, 121.8%, and 52.3%, in relation to T1, T3, and T4 treatments, respectively.

### 3.4. Mycorrhizal Dependency

**Figure 2:**
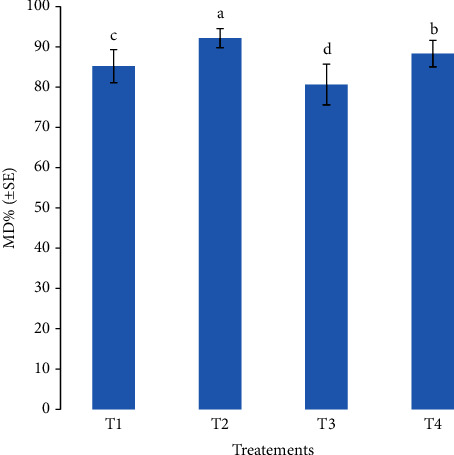
Comparison of mycorrhizal dependency (MD) among inoculation treatments. Treatments: T1: inoculation with a consortium with indigenous arbuscular mycorrhizal fungi (AMF); T2: dual inoculation with a consortium of AMF and *Bacillus subtilis*; T3: single inoculation with *Rhizophagus clarus*; T4: dual inoculation of *R. clarus* and *B. subtilis*; T5: single inoculation with *B. subtilis*; T6: negative control (noninoculated and not fertilized); T7: positive control (chemical fertilizer). Mean values of MD % followed by different letters indicate significant differences among the treatments after post hoc Tukey tests (*P* < 0.05).

Among studied host plants, *A. cepa* showed to have significantly higher mycorrhizal dependence than *L. esculentum* and *C. pepo* (Figure 1). Additionally, significant variations in mycorrhizal dependence were found between the studied treatments. Compared to the single inoculation of T1 (85.2%) or T3 (80.6%), the plants were more dependent on the dual inoculations of T2 (92.1%) and T4 (88.2%) (Figure 2).

### 3.5. Correlation between the Parameters

**Figure 3:**
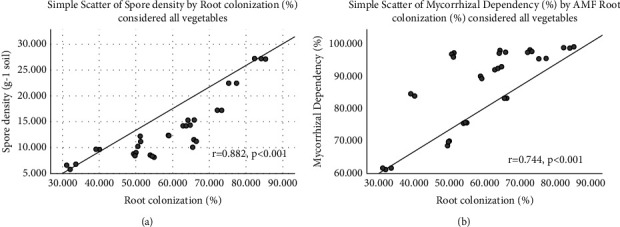
Correlation between AMF root colonization with spore density (a) and with mycorrhizal dependency (b). For correlations, analysis data of all studied species and treatments (see plant species and treatments at Tables 3 and 4) were considered.

Pearson correlation analysis between the parameters in each studied plant species was carried out to demonstrate the potential of inoculums and the growth responses (Table 5). The results revealed that strong and positive correlations were obtained between the plant growth parameters (*p* < 0.01). Total root colonization and plant height (*r* = 0.574) and TC and root height (*r* = 0.618) in *A. cepa* were shown to be strongly and positively correlated with one another. Very significant and positive correlations were also found between the TC and spore density (*r* = 0.882, *p* < 0.001) and between TC and mycorrhizal dependency (*r* = 0.744, *p* < 0.001), when all studied plant species were considered (Figure 3).

## 4. Discussion

The current study was focused on evaluating the effects of single and co-inoculation of indigenous AMF and a PGPR, *B. subtilis* ALCR46 strain indigenous from Ethiopia, on the growth of some economically important crop plants (*L. esculentum*, *A. cepa*, and *C. pepo*), which were grown under a soil low in fertility. The results showed that, except from the single inoculation of *A. cepa* with *B. subtilis* (T5), in which no increases in growth were found, both single and dual inoculation of *L. esculentum* and *C. pepo* significantly increased all growth parameters compared to noninoculated plants (T6). A previous study reported by Gupta et al. [31] also indicated that single inoculation of *A. cepa* with *Bacillus licheniformis* and *Pseudomonas fluorescens* showed no significant effect on most of the shoot and root growth parameters studied, and also showed an inhibitory growth effect on bulbs. They suggested that the antimicrobial properties of *A. cepa* in its root exudates, which may exist in the rhizosphere, could probably suppress the strain's growth and colonization.

Our study demonstrates that co-inoculation with the consortium of indigenous AMF + *B. subtilis* (T2) generally greatly boosted plant development, with outcomes comparable to those attained with plants that chemically fertilized (T7) (Table 3). This finding is quite consistent with observation made by Sreedhar and Mohan [32], who reported that co-inoculation with AMF and PGPR was not significantly different from those found in commercial chemical fertilizer. Moreover, other study [33] showed that the co-inoculation under field conditions with the endophytic fungi *Exserohilum rostratum* NMS1.5, and the AMF *Glomus etunicatum* UDCN52867, reached and even increased the growth and yields of sunchoke (*Helianthus tuberosus*) compared to a chemical fertilizer. This suggests that both AMF and PGPR indigenous to Ethiopian soils appear to be good candidates for use as bio-inoculants, which would reduce the use of chemical fertilizers, thereby reducing costs and environmental impact.

Additionally, in this study, all plants displayed higher growth performance when co-inoculated with the consortium of AMF and *B. subtilis* (T2) compared to the single inoculations (T1, T3, and T5). This suggests compatibility and synergism between AMF and the PGPR in enhancing the growth and biomass of host plants. Timonen et al. [34] evaluated the plant growth-promoting effects of inoculating *Rhizophagus irregularis* and/or *Bacillus amyloliquefaciens* in autoclaved substrates. They found that dual inoculation with *B. amyloliquefaciens* and *R. irregularis* resulted in the greatest increase in shoot weight and photosynthetic efficiency compared to a single inoculation. Co-inoculation effects of AMF and PGPR inoculation on plant growth performance compared to single inoculation with either of them have been reported elsewhere [35–37]. Overall, the experiment's findings demonstrated the two strains' compatibility and synergy in promoting growth and biomass yield. For example, a study by Awasthi et al. [35] in India, aimed to determine the effect of AMF species and two free-living nitrogen-fixing bacteria inoculated alone or in combinations with *B. subtilis* and the AMF *G. mosseae*, showed increased plant growth in inoculated than uninoculated control plants.

In this study, single inoculation with AMF both in consortium (T1) and of the individual strain *Rhizophagus clarus* (T3), respectively, showed to increase the growth of the host plants. This was expected and agrees with reports of Carrillo et al. [38] who showed that the tomato hybrid “El Cid” inoculated with the AMF *R. intraradices* significantly increased plant height, chlorophyll content, and root mycorrhizal colonization, in comparison with the noninoculated plants (Table 3). However, in our study, the single inoculation with the bacteria (T5 treatment) did not show significant growth response with respect to the control (noninoculated) plants. This suggests that *B. subtilis* ALCR46 strain seems not to be efficient to increase the growth of the plants studied. However, roots of plants co-inoculated with AMF + *B. subtilis* resulted in increases in height and weight but also showed significantly higher AMF colonization and spore density than treatment with single inoculation, indicating that AMF colonization under studied conditions was probably improved by the support of *B. subtilis* (Table 4).

Singh et al. [39] found increases in AMF root colonization and spore density after co-inoculation of *Coleus barbatus* with a native *Pseudomonas monteilii* strain and the AMF *Glomus fasciculatum* grown under organic field conditions. Marulanda-Aguirre et al. [40] also found that *Bacillus megaterium* co-inoculated with the AMF *G. intraradices* increased the percentage of mycorrhizal root length of *Lactuca sativa* plants compared to the single inoculation with *G. intraradices*. Our results reveal that the *B. subtilis* strain ALCR46 supports the growth of host plants supplied by the AMF (both the consortium and the single-strain *Rhizophagus clarus*) in a synergistic manner. Likewise, it also appears that *B. subtilis* ALCR46 acted as a “mycorrhizal helper bacterium” (MHB), as evidenced by increased spore proliferation in the substrate and colonization of host plant roots. Ramasamy et al. [41] reported that MHB stimulates AM propagule germination, hyphal growth, and root colonization. However, *ad hoc* studies (*in vitro* and in plant) should be conducted to confirm this hypothesis.

We also evaluated the growth-promoting ability of the AMF consortium treatment (T1) (inocula composed by the AMF spore mixture plus accompanying microflora) in comparison with that of the single AMF *Rhizophagus clarus* (T3). Consequently, plants infected with the consortium showed greater growth responses. Interestingly, better growth responses were found in plants co-inoculated with the AMF consortium + the bacteria (T2) compared to single *R*. *clarus* (T3), or dual inoculation with *R*. *clarus* + the bacteria (T4) (Table 3). This suggests that *B. subtilis* ALCR46 may create synergistic relationships with various AMF communities to encourage the development of the hosting plants. Likewise, He et al. [42] evaluated how the AMF composition affects the growth and nutrient acquisition of *Broussonetia papyrifera* (a woody shrub) and *Bidens pilosa* (an herbaceous plant) growing in pots in limestone soil from a Karst Area, China. They concluded that AM fungal associations increased plant growth and nutrient absorption and that, in general, inoculation with mixed AMF enhanced biomass and nutrient acquisition more than a single AM fungal inoculation. Additionally, Jansa et al. [43] reported that *Allium porrum* colonized by a mixture of AMF species acquired more P than plants inoculated with two AMF species separately. Direct evidence is provided for functional complementarity among species within the AMF community colonizing a single root system. In this respect, the inoculation with AMF consortia, which co-evolve in local ecological niches, is more robust and sustaining than inoculation with a single species [44].

In this study, the plant species *A. cepa* showed to be more dependent on AMF than the others studied host plants (Figure 1). The shape of the plant root system is associated with mycorrhizal dependence to some extent. It is probably that, because *Allium* species has a coarse root structure without root hairs, the studied species showed to be highly responsive to mycorrhizae formation [45]. There is evidence that genetic also has a role in how onions respond to mycorrhizal symbiosis [46]. These plants are frequently obligatory mycorrhizal crops that cannot finish their life cycle without AMF.

Positive and significant correlations (*p* < 0.05) between AM root colonization with AM spore density and mycorrhizal dependency of the studied plan species were found (Figure 3). A previous report by Sawant and Bhale [47] demonstrated that a Pearson correlation between the percentage of root colonization and spore density in the rhizosphere soil showed that a strong positive correlation was observed in Chilli (*r* = 0.799) and Brinjal (*r* = 0.899). Comparable to other reports, this study also showed that percent of AMF root colonization was positively correlated with total dry biomass, root height, and shoot height, respectively (Table 5), indicating that likely this growth benefit is due to the large extracellular hyphae AMF network, which transports water and nutrients to roots, increasing their absorption range and helping plants in the low nutrient soil [48].

## 5. Conclusions

This study shows evidence that the combination of a consortium with AMF plus *Bacillus subtilis* ALCR46, both native to Ethiopian soils, would be promising candidates for the formulation of biofertilizers that would provide nutrients and favor the growth of agriculturally important crops, reducing costs in synthetic fertilization and in environmental impact. The following studies should be oriented, on the one hand, to the taxonomic characterization of the species that belong to the consortium of AMF and, on the other hand, to the evaluation of the PGPM potential of the AMF and *Bacillus subtilis* combination in nonsterile soils and/or under field conditions [49, 50].

## Figures and Tables

**Table 1:** AMF spore density and physico-chemical parameters of soil samples from rhizosphere of acacia trees.

Sampling area	Acacia sp.	pH	TN (%)	AP (ppm)	OC (%)	Sand (%)	Clay (%)	Silt (%)	Class of texture	SD (g^−1^ soil)
Sululta	*A. abyssinica*	6.8	0.2	13.5	1.0	32	36	32	Clay loam	10.5 ± 0.3d

Batu	*A. saligna*	6.9	0.1	12.8	1.7	56	4	40	Sandy loam	18.1 ± 0.1a
	*A. seyal*	6.6	0.1	6.3	2.1	62	4	34	Sandy loam	18.8 ± 0.0a
	*A. tortilis*	7.3	0.1	11.1	1.6	58	6	36	Sandy loam	13.7 ± 0.0c

Bishoftu	*A. saligna*	7.0	0.1	12.7	0.8	50	17	33	Loam	14.4 ± 0.0c
	*A. seyal*	7.1	0.1	10.1	0.9	66	6	28	Sandy loam	16.6 ± 0.1b
	*A. tortilis*	7.1	0.1	9.6	1.6	50	14	36	Sandy loam	10.4 ± 0.1 d

AP: available phosphorus; TN: total nitrogen; OC: organic carbon; ppm: parts per million.

**Table 2:** Arbuscular mycorrhizal fungi spore density and percentage of root colonization of the trap cultures.

Sampling area	Source of the soil inoculum	AMF spore density (g^−1^ of soil)	AM colonization		
			AC (%)	VC (%)	TC (%)
Sululta	*A. abyssinica*	18.7 ± 0.4e	25.2 ± 0.0e	14.6 ± 0.1d	56.9 ± 0.2e

Batu	*A. saligna*	27.3 ± 0.0b	28.7 ± 0.2d	21 ± 0.6bc	76 ± 0.7b
	*A. seyal*	32.4 ± 0.2a	45.7 ± 0.8a	25.6 ± 0.4a	94.9 ± 0.4a
	*A. tortilis*	22.4 ± 0.0d	34.4 ± 0.4c	19.9 ± 0.4c	62.9 ± 1d

Bishoftu	*A. saligna*	25.6 ± 0.1c	27.3 ± 0.2d	15.1 ± 0.2d	67. ± 0.6c
	*A. seyal*	28.4 ± 0.1b	41.7 ± 0.4b	27.3 ± 0.4a	79 ± 1.1b
	*A. tortilis*	21.8 ± 0.1d	28.4 ± 0.4d	22.5 ± 0.4b	61 ± 0.5d

AC: arbuscular colonization; VC: vesicular colonization; TC: total colonization. Mean values followed by the same letter are not significantly different at *P* < 0.05. Mean ± standard error.

**Table 3:** Effect of inoculation and chemical fertilizer on growth characteristics of *L. esculentum*, *A. cepa,* and *C. pepo*.

Treatments	Plants parameters						
	SH (cm/p)	RH (cm/p)	NB (n/p)	SFW (g/p)	RFW (g/p)	SDW (g/p)	RDW (g/p)
*L. esculentum*							
T1	52.3 ± 0.1c	19.4 ± 0.3c	13 ± 0.0bc	28.6 ± 0.1c	4.4 ± 0.1c	6.0 ± 0.2c	1.0 ± 0.0c
T2	61.4 ± 0.3ab	25.9 ± 0.1a	14.3 ± 0.3ab	38.9 ± 0.1a	7.2 ± 0.3a	12.7 ± 0.1a	2.7 ± 0.0a
T3	50.3 ± 0.4c	16.8 ± 0.3d	13 ± 0.0bc	24 ± 0.1d	3 ± 0.0d	4 ± 0.0d	0.7 ± 0.0d
T4	57.8 ± 0.1b	22.8 ± 0.4b	13.6 ± 0.3ab	31.4 ± 0.4b	5.4 ± 0.12b	7.7 ± 0.1b	1.9 ± 0.0b
T5	44.2 ± 2d	14.6 ± 0.2e	11.6 ± 0.3cd	21.0 ± 0.6e	1.9 ± 0.06e	2 ± 0.0e	0.1 ± 0.0e
T6	29.2 ± 1.3e	10.7 ± 0.3f	10.3 ± 0.3d	15.4 ± 0.6f	1 ± 0.1f	0.7 ± 0.0f	0.0 ± 0.0f
T7	65.1 ± 0.1a	26.9 ± 0.5a	14.6 ± 0.3a	39.7 ± 0.2a	7.5 ± 0.2a	12.1 ± 0.1a	2.6 ± 0.1a

*A. cepa*							
T1	50.4 ± 0.6c	12.2 ± 0.2bc	3.6 ± 0.3ab	11.9 ± 0.2b	0.8 ± 0.0b	0.7 ± 0.0b	0.04 ± 0.0b
T2	57.9 ± 0.1b	15.2 ± 0.3a	5 ± 0.0a	15.1 ± 0.3a	1.6 ± 0.0a	1.4 ± 0.0a	0.06 ± 0.0a
T3	45.5 ± 0.2d	11.7 ± 0.2c	3.6 ± 0.3ab	9.2 ± 0.2c	0.4 ± 0.0c	0.6 ± 0.0c	0.02 ± 0.0c
T4	52.4 ± 0.7c	13.3 ± 0.3b	4.6 ± 0.3a	13 ± 0.4b	1.1 ± 0.1b	0.8 ± 0.0b	0.04 ± 0.0b
T5	20.6 ± 0.9e	4.6 ± 0.4d	2.6 ± 0.3b	1.2 ± 0.0d	0.0 ± 0.0d	0.04 ± 0.0d	0.01 ± 0.0d
T6	18.5 ± 0.3e	4.3 ± 0.4d	2.3 ± 0.3b	1.1 ± 0.0d	0.03 ± 0.0d	0.03 ± 0.0d	0.01 ± 0.0d
T7	59.4 ± 0.7a	15.2 ± 0.2a	5 ± 0.0a	16.5 ± 0.6a	1.9 ± 0.0a	1.5 ± 0.0a	0.06 ± 0.0a

*C. pepo*							
T1	66.8 ± 0.5c	8 ± 3bc	8.3 ± 0.3ab	48.2 ± 0.1bc	3.4 ± 0.2b	7.9 ± 0.1c	1.0 ± 0.0c
T2	71.9 ± 0.8ab	10.8 ± 0.3a	9 ± 0.0a	54 ± 0.3a	5.0 ± 0.1a	14 ± 0.0a	2.2 ± 0.0a
T3	64.8 ± 0.5c	6.2 ± 0.2cd	7.3 ± 0.3b	45.5 ± 0.9c	2.1 ± 0.1c	6.7 ± 0.0d	0.8 ± 0.0c
T4	68.7 ± 0.2bc	9.5 ± 0.3ab	8.6 ± 0.3ab	52.6 ± 0.2ab	3.8 ± 0.2b	9.8 ± 0.0b	1.4 ± 0.0b
T5	58.7 ± 1.1d	4.9 ± 0.1de	5.6 ± 0.3c	38.9 ± 0.7d	1.4 ± 0.0cd	5.0 ± 0.0e	0.5 ± 0.0e
T6	46.1 ± 1.5e	3.7 ± 0.1e	5.3 ± 0.3c	28.4 ± 0.1e	0.9 ± 0.0d	2.7 ± 0.0f	0.3 ± 0.0e
T7	73 ± 0.5a	11.1 ± 0.7a	9.3 ± 0.3a	57.3 ± 1.1a	5.4 ± 0.2a	14.2 ± 0.2a	2.2 ± 0.0a

T1: inoculation with a consortium with AMF; T2: dual inoculation with a consortium of AMF and *Bacillus subtilis*; T3: single inoculation with *Rhizophagus clarus*; T4: dual inoculation of *R. clarus* and *B. subtilis*; T5: single inoculation with *B. subtilis*; T6: negative control (noninoculated and not fertilized); T7: positive control (chemical fertilizer). SH—shoot height, RH—root height, NB—number of branches, SFW—fresh weight of shoot, RFW—fresh weight of root, SDW—dry weight of shoot, RDW—dry weight of root. The same letter in the row indicates not significantly different values based on one-way ANOVA and Tukey's honestly significant difference (HSD) post hoc test (*p* < 0.05).

**Table 4:** Effects of single and co-inoculation of AMF and PGPR inoculums on the rate of total AMF root colonization and spore density of *L. esculentum*, *A. cepa*, and *C. pepo.*

Treatments	*L. esculentum*			*A. cepa*			*C. pepo*		
	TC (%)		SD g^−1^ soil	TC (%)		SD g^−1^ soil	TC (%)		SD g^−1^ soil
	After 35 d	After 60 d	After 60 d	After 35 d	After 60 d	After 60 d	After 35 d	After 60 d	After 60 d
T1	16.6 ± 0.3c	59.0 ± 0.1c	12.2 ± 0.0c	18.6 ± 0.3c	64.7 ± 0.5c	15.3 ± 0.0c	12.2 ± 0.5c	49.7 ± 0.1c	8.5 ± 0.1b
T2	28.3 ± 0.5a	76.6 ± 0.6a	22.4 ± 0.0a	36.6 ± 0.7a	83.9 ± 0.8a	27.1 ± 0.0a	23.6 ± 0.3a	65.6 ± 0.2a	10.9 ± 0.4a
T3	7.6 ± 1.9d	39.3 ± 0.3d	9.6 ± 0.0d	11.3 ± 0.5d	50.9 ± 0.2d	11.2 ± 0.0d	2.9 ± 0.3d	32.1 ± 0.7d	6.4 ± 0.3c
T4	19.2 ± 0.5b	63.7 ± 0.5b	14.2 ± 0.0b	23.2 ± 0.6b	72.8 ± 0.3b	17.2 ± 0.0b	15.5 ± 2.8b	54.4 ± 0.3b	8.3 ± 0.1b
T5		—	—		—	—		—	—
T6		—	—		—	—		—	—
T7		—	—		—	—		—	—

T1: inoculation with a consortium with indigenous AMF; T2: dual inoculation with a consortium of AMF and *Bacillus subtilis*; T3: single inoculation with *Rhizophagus clarus*; T4: dual inoculation of *R. clarus* and *B. subtilis*; T5: single inoculation with *B. subtilis*; T6: negative control (noninoculated and not fertilized); T7: positive control (chemical fertilizer). TC: total colorization; SD: spore density. Mean values followed by the same letter are not significantly different at *p*=0.05. Mean ± standard error.

**Table 5:** Pearson correlation coefficients between the parameters in each selected vegetable.

	SH	RH	NB	SDW	RDW	TDW	TC	SD
*Lycopersicum esculentum*								
SH	1	0.959^*∗∗*^	0.958^*∗∗*^	0.905^*∗∗*^	0.899^*∗∗*^	0.906^*∗∗*^	0.475^*∗*^	0.489^*∗*^
RH	0.959^*∗∗*^	1	0.930^*∗∗*^	0.975^*∗∗*^	0.972^*∗∗*^	0.976^*∗∗*^	0.465^*∗*^	0.500^*∗*^
NB	0.958^*∗∗*^	0.930^*∗∗*^	1	0.893^*∗∗*^	0.884^*∗∗*^	0.893^*∗∗*^	0.487^*∗*^	0.509^*∗*^
SDW	0.905^*∗∗*^	0.975^*∗∗*^	0.893^*∗∗*^	1	0.988^*∗∗*^	1.000^*∗∗*^	0.457^*∗*^	0.522^*∗*^
RDW	0.899^*∗∗*^	0.972^*∗∗*^	0.884^*∗∗*^	0.988^*∗∗*^	1	0.992^*∗∗*^	0.473^*∗*^	0.528^*∗*^
TDW	0.906^*∗∗*^	0.976^*∗∗*^	0.893^*∗∗*^	1.000^*∗∗*^	0.992^*∗∗*^	1	0.461^*∗*^	0.524^*∗*^
TC	0.475^*∗*^	0.465^*∗*^	0.487^*∗*^	0.457^*∗*^	0.473^*∗*^	0.461^*∗*^	1	0.978^*∗∗*^
SD	0.489^*∗*^	0.500^*∗*^	0.509^*∗*^	0.522^*∗*^	0.528^*∗*^	0.524^*∗*^	0.978^*∗∗*^	1

*Allium cepa*								
SH	1	0.991^*∗∗*^	0.877^*∗∗*^	0.952^*∗∗*^	0.949^*∗∗*^	0.953^*∗∗*^	0.574^*∗∗*^	0.556^*∗∗*^
RH	0.991^*∗∗*^	1	0.876^*∗∗*^	0.947^*∗∗*^	0.944^*∗∗*^	0.947^*∗∗*^	0.618^*∗∗*^	0.599^*∗∗*^
NB	0.877^*∗∗*^	0.876^*∗∗*^	1	0.890^*∗∗*^	0.878^*∗∗*^	0.890^*∗∗*^	0.501^*∗*^	0.544^*∗*^
SDW	0.952^*∗∗*^	0.947^*∗∗*^	0.890^*∗∗*^	1	0.981^*∗∗*^	1.000^*∗∗*^	0.456^*∗*^	0.536^*∗*^
RDW	0.949^*∗∗*^	0.944^*∗∗*^	0.878^*∗∗*^	0.981^*∗∗*^	1	0.982^*∗∗*^	0.506^*∗*^	0.593^*∗∗*^
TDW	0.953^*∗∗*^	0.947^*∗∗*^	0.890^*∗∗*^	1.000^*∗∗*^	0.982^*∗∗*^	1	0.458^*∗*^	0.538^*∗*^
TC	0.574^*∗∗*^	0.618^*∗∗*^	0.501^*∗*^	0.456^*∗*^	0.506^*∗*^	0.458^*∗*^	1	0.930^*∗∗*^
SD	0.556^*∗∗*^	0.599^*∗∗*^	0.544^*∗*^	0.536^*∗*^	0.593^*∗∗*^	0.538^*∗*^	0.930^*∗∗*^	1

*Cucurbita pepo*								
SH	1	0.900^*∗∗*^	0.871^*∗∗*^	0.892^*∗∗*^	0.840^*∗∗*^	0.885^*∗∗*^	0.543^*∗*^	0.539^*∗*^
RH	0.900^*∗∗*^	1	0.915^*∗∗*^	0.955^*∗∗*^	0.960^*∗∗*^	0.959^*∗∗*^	0.508^*∗*^	0.473^*∗*^
NB	0.871^*∗∗*^	0.915^*∗∗*^	1	0.886^*∗∗*^	0.892^*∗∗*^	0.890^*∗∗*^	0.568^*∗∗*^	0.553^*∗∗*^
SDW	0.892^*∗∗*^	0.955^*∗∗*^	0.886^*∗∗*^	1	0.981^*∗∗*^	0.999^*∗∗*^	0.427	0.401
RDW	0.840^*∗∗*^	0.960^*∗∗*^	0.892^*∗∗*^	0.981^*∗∗*^	1	0.987^*∗∗*^	0.453^*∗*^	0.418
TDW	0.885^*∗∗*^	0.959^*∗∗*^	0.890^*∗∗*^	0.999^*∗∗*^	0.987^*∗∗*^	1	0.433^*∗*^	0.405
TC	0.543^*∗*^	0.508^*∗*^	0.568^*∗∗*^	0.427	0.453^*∗*^	0.433^*∗*^	1	0.991^*∗∗*^
SD	0.539^*∗*^	0.473^*∗*^	0.553^*∗∗*^	0.401	0.418	0.405	0.991^*∗∗*^	1

SH: shoot height; RH: root height; NB: number of branches; SDW: shoot dry weight; RDW: root dry weight; TDW: total dry weight: TC: total root mycorrhizal colonization; SD: spore density. ^*∗∗*^Correlation is significant at the 0.01 level (2-tailed). ^*∗*^Correlation is significant at the 0.05 level (2-tailed).

## Data Availability

The data used to support the findings of the study are available from the corresponding author upon request.
